# AMPK-Dependent Mechanisms but Not Hypothalamic Lipid Signaling Mediates GH-Secretory Responses to GHRH and Ghrelin

**DOI:** 10.3390/cells9091940

**Published:** 2020-08-21

**Authors:** María J. Vázquez, Marta G. Novelle, Francisca Rodríguez-Pacheco, Ricardo Lage, Luis Varela, Miguel López, Leonor Pinilla, Manuel Tena-Sempere, Carlos Diéguez

**Affiliations:** 1 CIBER Fisiopatología de la Obesidad y Nutrición (CIBERobn), Instituto de Salud Carlos III, 15782 Santiago de Compostela, Spain; bc2vavim@uco.es (M.J.V.); paqui.endocrino@gmail.com (F.R.-P.); rlagef@gmail.com (R.L.); luis.varela@yale.edu (L.V.); m.lopez@usc.es (M.L.); bc1pijul@uco.es (L.P.); 2 Department of Cell Biology, Physiology and Immunology, School of Medicine, University of Córdoba—Instituto Maimónides de Investigaciones Biomédicas (IMIBIC), 14004 Córdoba, Spain; 3 Centro singular de Investigación en Medicina Molecular y Enfermedades Crónicas (CiMUS), Universidad de Santiago de Compostela, Health Research Institute of Santiago de Compostela (IDIS), 15782 Santiago de Compostela, Spain

**Keywords:** AMPK, GH, GHRH, ghrelin, hypothalamic signaling

## Abstract

GH (growth hormone) secretion/action is modulated by alterations in energy homeostasis, such as malnutrition and obesity. Recent data have uncovered the mechanism by which hypothalamic neurons sense nutrient bioavailability, with a relevant contribution of AMPK (AMP-activated protein kinase) and mTOR (mammalian Target of Rapamycin), as sensors of cellular energy status. However, whether central AMPK-mediated lipid signaling and mTOR participate in the regulation of pituitary GH secretion remains unexplored. We provide herein evidence for the involvement of hypothalamic AMPK signaling, but not hypothalamic lipid metabolism or CPT-1 (carnitine palmitoyltransferase I) activity, in the regulation of GH stimulatory responses to the two major elicitors of GH release in vivo, namely GHRH (growth hormone–releasing hormone) and ghrelin. This effect appeared to be GH-specific, as blocking of hypothalamic AMPK failed to influence GnRH (gonadotropin-releasing hormone)-induced LH (luteinizing hormone) secretion. Additionally, central mTOR inactivation did not alter GH responses to GHRH or ghrelin, nor this blockade affected LH responses to GnRH in vivo. In sum, we document here for the first time the indispensable and specific role of preserved central AMPK, but not mTOR, signaling, through a non-canonical lipid signaling pathway, for proper GH responses to GHRH and ghrelin in vivo.

## 1. Introduction

In addition to stimulating body growth, GH (growth hormone) plays an important role in metabolism. In turn, various products of intermediary metabolism, such as glucose, free fatty acids, dietary proteins and amino acids act on both the hypothalamus and the anterior pituitary to control the function of GH-producing cells [1]. Alterations in energy homeostasis, such as malnutrition and obesity, exert marked effects on GH secretion and/or its actions at target tissues. Therefore, metabolic substrates and GH secretion influence each other in a process that can be considered as an integrated response of the overall regulation of feeding and fasting in order to maintain adequate energy homeostasis and fat mass.

Data gleaned over the last few years have uncovered the mechanism by which cells sense nutrient bioavailability. These include specific transmembrane transporters as well as membrane receptors, which directly or indirectly influence the so-called *cellular energy sensors* that in turn modify different intracellular signal pathways. In this regard, work carried out throughout the last decade has highlighted the importance of AMPK (AMP-activated protein kinase) [2,3,4,5,6] and mTOR (mammalian Target of Rapamycin) [7,8,9,10] in the hypothalamic control of energy and metabolic homeostasis, frequently operating and being regulated in a reciprocal manner [11,12]. Thus, both cellular sensors are regulated by fasting and feeding, and their activity is linked to changes in food intake and body weight homeostasis. Noteworthy, they appear to play an essential transducing role at the hypothalamic level mediating the effects in terms of energy and metabolic homeostasis of different neuropeptides, peripheral hormones and several nutrients [6,13,14].

Because of the important interrelationship among energy homeostasis, lipid metabolism and GH secretion, we hypothesized that the cellular sensors, AMPK and mTOR, could be involved in the neuro-regulation of GH secretion. This study was prompted by our studies showing that ghrelin, a peptide known as a potent GH-secretagogue, exerts its orexigenic effect through a mechanism involving the activation of hypothalamic AMPK and inactivation of acetyl-CoA carboxylase (ACC) and fatty acid synthase (FAS), which result in decreased hypothalamic levels of malonyl-CoA, increased carnitine palmitoyl-transferase 1 (CPT1) activity and mitochondrial production of reactive oxygen species (ROS) [4,15,16,17,18]. In addition, ghrelin has been shown to activate hypothalamic mTOR signaling, while inactivation of central mTOR by rapamycin attenuated ghrelin’s orexigenic effect [15], and caused inhibition of the gonadotropic axis and puberty; this period is one of the major determinants of GH secretory status [19,20]. Likewise, central AMPK signaling has been shown to modulate pubertal timing, since it operates as a conduit for the inhibitory actions of conditions of negative energy balance on puberty onset in female rodents [21].

In the above context, this study aimed to investigate whether GH secretion, either in basal conditions or after growth hormone–releasing hormone (GHRH) or ghrelin stimulation, is modulated by changes in central AMPK or mTOR signaling. To this end, circulating GH levels and stimulated responses were assessed following the inactivation of hypothalamic AMPK or one of its major downstream factors, namely CPT-1. In addition, we evaluated the effect of mTOR blockade on GH secretion in basal and stimulated conditions. In order to assess the specificity of the effects of such manipulations on GH secretion, similar experiments were conducted assaying LH (luteinizing hormone) responses to GnRH (gonadotropin-releasing hormone) after selective blockade of AMPK or mTOR signaling pathways.

## 2. Material and Methods

### 2.1. Animals and Experimental Procedure

Young adult male Sprague–Dawley rats (6–8 weeks old; body weight 200–250 g) were housed in a 12-h light: 12-h darkness cycle in a temperature- and humidity-controlled room. Chronic intracerebro-ventricular (icv) and intravenous (iv) cannulae were implanted in the animal under ketamine/xylazine anesthesia, as described previously [22]. After surgery, the animals were placed directly in isolation test chambers for 5 days and were given free access to regular rat chow and tap water. Thereafter, the animals continued to have food available ad libitum. On the day of the experiment, blood samples (0.3 mL) were withdrawn at the designed times using protocols of icv and iv administration in conscious and freely-moving rats. The experimental protocols were approved by the University of Santiago de Compostela ethics committee, and experiments were performed in agreement with the rules of laboratory animal care and international law on animal experimentation.

As a general procedure for the different experiments, the animals (*n* = 8–9 rats/group; none of them was used for more than a single experiment) received a first bolus through the icv cannulae (for central blockade of AMPK or mTOR routes) and a second one 10 min later through the iv route (time-0; for stimulation of GH secretion), followed by serial blood sampling. In detail, blood samples were taken 30 and 15 min before icv administration of vehicle, compound C (CC), etomoxir or rapamycin and 0, 5, 10, 15, 20, 30 and 45 min after iv administration of vehicle, GHRH (growth hormone-releasing hormone) or ghrelin. GnRH was purchased from Sigma-Aldrich, (St Louis, MO, USA). Human ghrelin and GHRH (human (GRF 1–29) amide) were supplied by Bachem (Bubendorf, Switzerland). DMSO used as solvent was provided by Sigma-Aldrich (St Louis, MO, USA). Other compounds used in this study are summarized in Table 1.

Using the same experimental design [21], doses and routes of administration, we have previously reported the effects of ghrelin, CC, etomoxir and rapamycin on food intake as well as their effects on hypothalamic pAMPK (CC), CPT-1c (etomoxir) and mTOR (rapamycin) [15,18].

### 2.2. Experimental Design

Exp. 1: Effect of inactivation of central AMPK on GH responses to GHRH and ghrelin.

The profile of dynamic GH response to GHRH or ghrelin after impairment of hypothalamic AMPK was evaluated. The experimental groups were assigned as follows: VH (5 µL DMSO icv plus 200 µL saline iv), CC (10 µg compound C icv plus 200 µL saline iv), GHRH (5 µL DMSO icv plus 12 nmol/kg BW GHRH iv), CC+GHRH (10 µg compound C plus 12 nmol/kg BW GHRH iv), ghrelin (5 µL DMSO icv plus 12 nmol/kg BW ghrelin iv) or CC+ghrelin (10 µg compound C icv plus 12 nmol/kg BW ghrelin iv).

Exp. 2: Effect of central CPT1 inhibition on GH responses to GHRH and ghrelin.

The potential influence of central inactivation of CPT1 activity on GHRH- or ghrelin-induced GH secretion was determined. For this purpose, the following experimental groups were established: VH (5 µL DMSO icv plus 200 µL saline iv), ETOM (10 µg etomoxir icv plus 200 µL saline iv), GHRH (5 µL DMSO icv plus 12 nmol/kg BW GHRH iv) or ETOM+GHRH (10 µg etomoxir plus 12 nmol/kg BW GHRH iv), ghrelin (5 µL DMSO icv plus 12 nmol/kg BW ghrelin iv), ETOM+ghrelin (10 µg etomoxir icv plus 12 nmol/kg BW ghrelin iv). Using the same experimental approach, we have previously reported that etomoxir inhibits CPT1 [13,18].

Exp. 3: Effect of central mTOR inhibition on GH responses to GHRH and ghrelin.

The impact of the inactivation of hypothalamic mTOR on GH responses to ghrelin or GHRH was evaluated. The following experimental groups were studied: VH (5 µL DMSO icv plus 200 µL saline iv), RAPA (50 µg rapamycin icv plus 200 µL saline iv), GHRH (5 µL DMSO icv plus 12 nmol/kg BW GHRH iv) or RAPA+GHRH (50 µg rapamycin icv plus 12 nmol/kg BW GHRH iv), ghrelin (5 µL DMSO icv plus 12 nmol/kg BW ghrelin iv) and RAPA+ghrelin (50 µg rapamycin icv plus 12 nmol/kg BW ghrelin iv). Using the same experimental approach, we have previously reported that rapamycin inhibits mTOR activity [15].

Exp. 4: Effect of central AMPK or mTOR inhibition on LH responses to GnRH.

In order to evaluate the specificity of the GH effects observed in Exp. 1–3, analogous experiments were conducted assessing the effect of iv administration of GnRH (32.5 nmol/kg BW) on LH secretion following central inactivation of AMPK or mTOR signaling. Blood samples were collected in 15-min intervals. Using the same experimental approach, we have previously reported that rapamycin inhibits mTOR activity [20].

### 2.3. Hormone Assays

Serum hormone concentrations were determined by double-antibody RIAs using materials supplied by the National Hormone Pituitary Program (Dr AF Parlow, NIDDK National Hormone and Peptide Program; Torrance, CA, USA), as described previously [23]. GH levels were determined in a volume of 20 µL. Rat GH was labeled with ^125^I by the chloramine-T method. Values are expressed in terms of the GH reference preparation (GH-RP-2). The intra- and inter-assay coefficients of variation were 7% and 10% respectively, and the sensitivity of the assay was 250 pg/mL. Serum LH levels were determined in a volume of 25 µL. Rat LH-I-10 was labeled with ^125^I using Iodo-gen^®^ tubes, following the instructions of the manufacturer (Pierce, Rockford, IL, USA). Hormone concentrations were expressed using reference preparations LH-RP-3 as standards. Intra- and inter-assay coefficients of variation were <8% and 10%, respectively. The sensitivity of the assay was 75 pg/mL. For each experiment and each hormone, all samples were measured in the same assay. Accuracy of hormone determinations was confirmed by the assessment of rat serum samples of known hormone concentrations.

### 2.4. Statistical Analysis

Data are expressed as mean ± SEM. AUC (area under the curve) data were analyzed by two-way ANOVA repeated measures. In the case of hormone profiles, the data were analyzed by two-way ANOVA repeated measures followed by post hoc Bonferroni’s test. All analyses were assessed using PASW Statistics 18.0 software (SPSS Inc., Chicago, IL, USA). A value of *p* < 0.05 was considered as being significant.

## 3. Results

### 3.1. Blockade of Hypothalamic AMPK blunts GHRH- and Ghrelin-Stimulated GH Secretion

**Figure 1:**
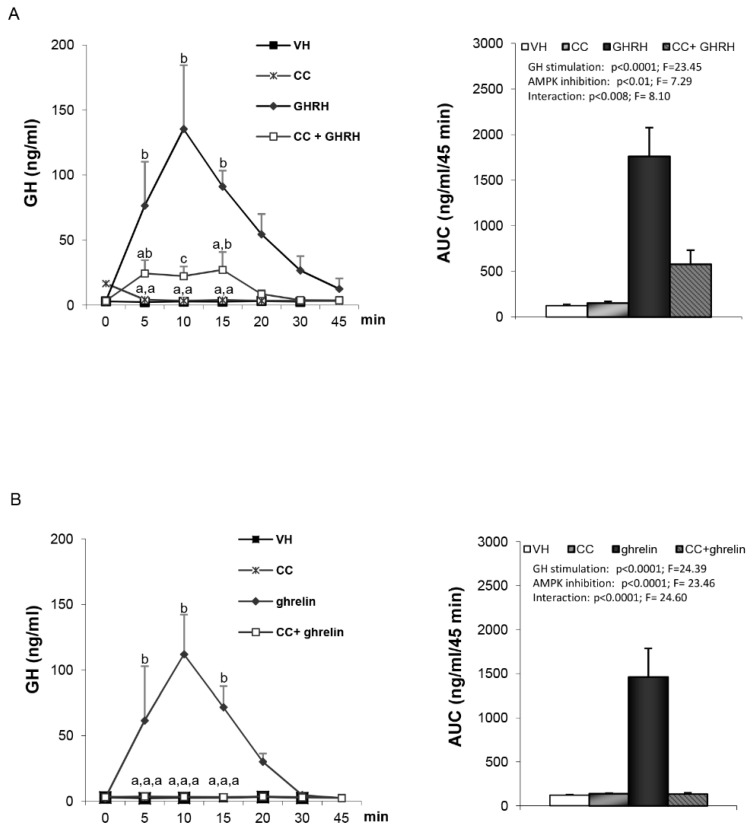
Serum growth hormone (GH) profile and area under the curve (AUC) over the study period (45 min) after administration of: (**A**) VH (5 µL DMSO icv plus 200 µL saline iv), CC (10 µg compound C icv plus 200 µL saline iv), growth hormone–releasing hormone (GHRH) (5 µL DMSO icv plus 12 nmol/kg GHRH iv), CC+GHRH (10 µg compound C plus 12 nmol/kg GHRH iv). (**B**) VH (5 µL DMSO icv plus 200 µL saline iv), CC (10 µg compound C icv plus 200 µL saline iv), ghrelin (5 µL DMSO icv plus 12 nmol/kg ghrelin iv) or CC + ghrelin (10 µg compound C icv plus 12 nmol/kg ghrelin iv). Administration of the GH-stimulating peptides took place after stabilization of the animals following the third sampling point (indicated as time 0). Values are given as the mean ± SEM. *p* < 0.05; two-way ANOVA repeated measures followed, in case of hormone profile, by a post hoc Bonferroni’s test (groups with different superscript letters are statistically different).

**Figure 2:**
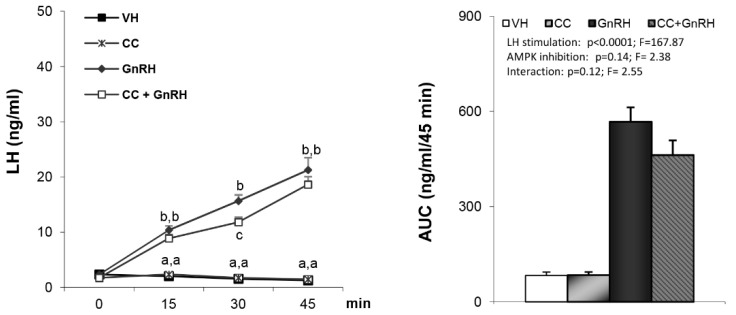
Levels of luteinizing hormone (LH) secretion and AUC after treatment with VH (5 µL DMSO icv plus 200 µL saline iv), CC (10 µg compound C icv plus 200 µL saline iv), gonadotropin-releasing hormone (GnRH) (5 µL DMSO icv plus 32.5 nmol/kg iv) or CC + GnRH (10 µg compound C plus 32.5 nmol/kg iv). Administration of the peptides took place after stabilization of the animals following the third sampling point (indicated as time 0). Values are given as the mean ± SEM. *p* < 0.05; two-way ANOVA repeated measures followed, in case of hormone profile, by a post hoc Bonferroni’s test (groups with different superscript letters are statistically different).

As expected, administration of either GHRH or ghrelin to vehicle-treated rats led to a clear increase in plasma GH levels (Figure 1A,B). GH responses to GHRH (Figure 1A) were markedly reduced in compound C pre-treated rats (AUC: 1761.16 ± 315.74 vs. 579.04 ± 153.61). Similar findings were observed following ghrelin induced GH secretion (Figure 1B), where compound C pre-treated rats exhibited an almost complete absence of GH responses, as assessed at different time-points (Figure 1B, left panel) and by AUC (1461.17 ± 326.97 vs. 135.3 ± 12.85) (Figure 1B, right panel). Taken together, these data demonstrate that the stimulatory effect of both GHRH and ghrelin on GH secretion is hypothalamic AMPK-dependent. This effect appears to be quite specific since LH responses to GnRH were unaffected by pretreatment with compound C (Figure 2).

### 3.2. Hypothalamic Lipid Metabolism does not Affect GHRH- or Ghrelin-Stimulated GH Secretion

**Figure 3:**
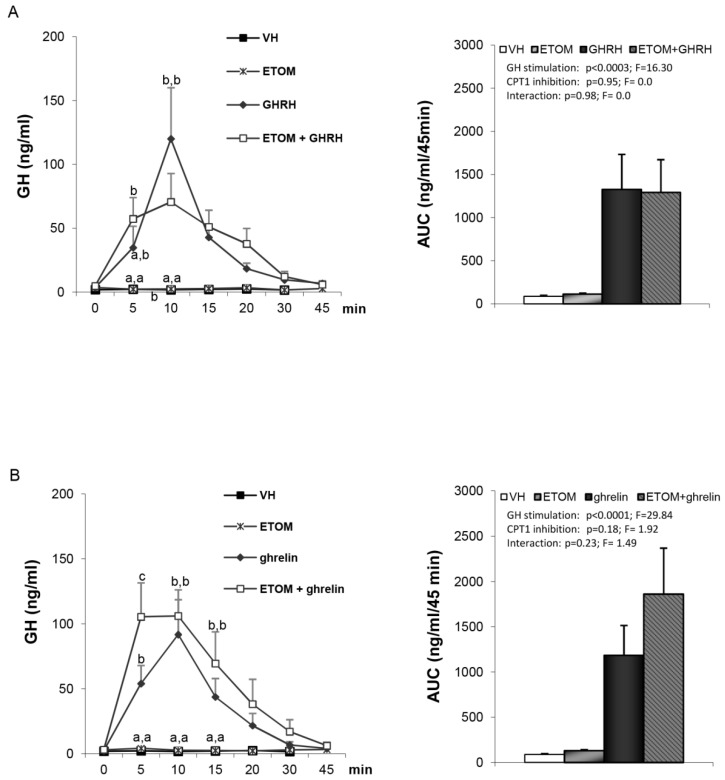
Serum GH profile and area under the curve (AUC) over the study period (45 min) after the administration of: Panel (**A**) VH (5 µL DMSO icv plus 200 µL saline iv), ETOM (10 µg etomoxir icv plus 200 µL saline iv), GHRH (5 µL DMSO icv plus 12 nmol/kg GHRH iv) or ETOM+GHRH (10 µg etomoxir plus 12 nmol/kg GHRH iv). Panel (**B**) VH (5 µL DMSO icv plus 200 µL saline iv), ETOM (10 µg etomoxir icv plus 200 µL saline iv), ghrelin (5 µL DMSO icv plus 12 nmol/kg ghrelin iv) or ETOM + ghrelin (10 µg etomoxir icv plus 12 nmol/kg ghrelin iv). Administration of the peptides took place after stabilization of the animals following the third sampling point (indicated as time 0). Values are given as the mean ± SEM. *p* < 0.05; two-way ANOVA repeated measures followed, in case of hormone profile, by a post hoc Bonferroni’s test (groups with different superscript letters are statistically different).

Since the orexigenic effect of ghrelin is exerted by AMPK through a signaling cascade involving hypothalamic lipid metabolism (ACC, FAS, malonyl CoA and CPT-1) and can be blocked by either inactivation of AMPK or by blocking CPT-1 [18], we tested whether impairment of CPT-1 activity with etomoxir, at a dose known to block the ghrelin-induced increase in food intake, could also blunt ghrelin- or GHRH-stimulated GH secretion. However, in contrast to previous data on food intake [18], we found that GH responses to either GHRH (Figure 3A) or ghrelin (Figure 3B) were similar in vehicle- or etomoxir-pretreated rats.

### 3.3. Hypothalamic mTOR is not Involved in GHRH- and Ghrelin-Stimulated GH Secretion

**Figure 4:**
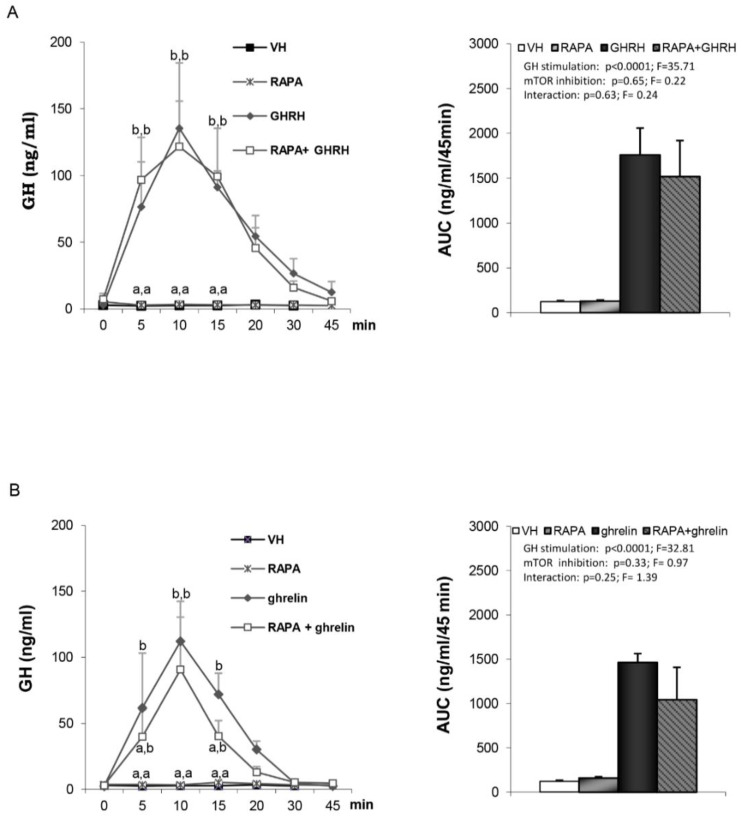
Serum GH profile and area under the curve (AUC) over the study period (45 min) after administration of: Panel (**A**) VH (5 µL DMSO icv plus 200 µL saline iv), RAPA (50 µg rapamycin icv plus 200 µL saline iv), GHRH (5 µL DMSO icv plus 12 nmol/kg GHRH iv) or RAPA+GHRH (50 µg rapamycin icv plus 12 nmol/kg GHRH iv). Panel (**B**) VH (5 µL DMSO icv plus 200 µL saline iv), RAPA (50 µg rapamycin icv plus 200 µL saline iv), ghrelin (5 µL DMSO icv plus 12 nmol/kg ghrelin iv) or RAPA + ghrelin (50 µg rapamycin icv plus 12 nmol/kg ghrelin iv). Administration of the peptides took place after stabilization of the animals following the third sampling point (indicated as time 0). Values are given as the mean ± SEM. *p* < 0.05; two-way ANOVA repeated measures followed, in case of hormone profile, by a post hoc Bonferroni’s test (groups with different superscript letters are statistically different).

**Figure 5:**
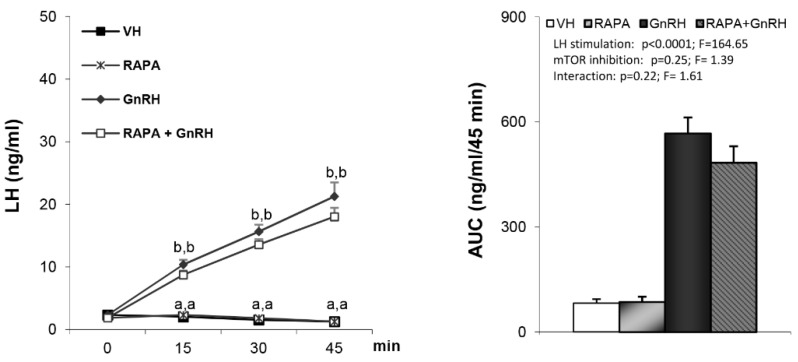
Levels of LH secretion and AUC after treatment; VH (5 µL DMSO icv plus 200 µL saline iv), RAPA (50 µg rapamycin icv plus 200 µL saline iv), GnRH (5 µL DMSO icv plus 32.5 nmol/kg iv) or RAPA + GnRH (50 µg rapamycin plus 32.5 nmol/kg iv). Administration of the peptides took place after stabilization of the animals following the third sampling point (indicated as time 0). Values are given as the mean ± SEM. *p* < 0.05; two-way ANOVA repeated measures followed, in case of hormone profile, by a post hoc Bonferroni’s test (groups with different superscript letters are statistically different).

It is well established that mTOR signaling at discrete hypothalamic neuronal populations plays an important role in energy homeostasis and has been shown to be a mediator of leptin’s anorexigenic effects [24,25]; a hormone that is also involved in the control of GH secretion [26]. Thus, we assessed whether central inactivation of mTOR by rapamycin may influence GH secretion. We found a similar response to either GHRH (Figure 4A) or ghrelin (Figure 4B) in vehicle- and rapamycin-treated rats. Likewise, GnRH-induced LH secretion was not affected by the inactivation of central mTOR signaling by rapamycin (Figure 5).

## 4. Discussion

The importance of hypothalamic AMPK in the regulation of different homeostatic processes has been highlighted by previous reports showing that AMPK is expressed in key hypothalamic nuclei, is regulated by fasting, increases feeding, plays an important role in central (hypothalamic) sensing of hypoglycemia and is regulated by different hormones and neurotransmitters, including ghrelin [6,27]. In fact, ghrelin exerts its orexigenic effect through an AMPK-dependent mechanism. However, even though the hypothalamus plays a major role in the control of anterior pituitary hormone secretion, the putative roles of AMPK in the control of the hypothalamus–pituitary axis remain unexplored. Moreover, from data gleaned during the last years, AMPK has emerged as a neuronal energy sensor exerting many of its biological effects through hypothalamic fatty acid metabolism. In this regard, malonyl-CoA has emerged as a key metabolic effector, with both CPT1a and CPT1c being part of the signaling pathway [28,29]. The relevance of this signaling pathways is highlighted by the following: (i) increased in hypothalamic malonyl-CoA and inhibition of CPT1 exerted a marked anorexigenic effect [30,31]; (ii) many signals involved in energy and metabolic homeostasis, including ghrelin, leptin, GLP-1 (glucagon-like peptide-1), BMP8b (bone morphogenetic protein 8b), thyroid hormones and estradiol, exert their effects on energy balance through this pathway [32,33,34]; (iii) the canonical pathway of AMPK(VMH)-CPT1c-SNS-BAT-has recently emerged as playing a key role for brown fat thermogenesis and browning of white adipose tissue [35]; and (iv) this signaling pathway is also involved in other neuroendocrine effects linked to axon growth, food preference, reproduction or central control of peripheral lipid and glucose homeostasis [21,36,37]. Taking into account the importance of energy status on in vivo GH secretion and the above features, we found it highly relevant to assess the role of hypothalamic AMPK-lipid metabolism signaling pathway in the control of GH secretion, in basal and stimulated conditions.

To our knowledge, our study is the first to demonstrate the functional involvement of hypothalamic AMPK in the control of GH secretion in vivo. It should be noted that we found that functional impairment of hypothalamic AMPK led to an almost complete absence of GH responses to GHRH or ghrelin, although both peptides were given at the maximal effective doses in terms of GH secretion. The observation that blocking of central AMPK signaling inhibits ghrelin-induced GH secretion was not completely unexpected in the light of our previous studies showing that it also prevents ghrelin orexigenic effect [18]. Taking into account that ghrelin exerts their effects on GH secretion through a complex mechanism at the hypothalamic level involving the increase GHRH secretion, the decrease of the somatostatinergic tone and the possible release of as yet unknown factor(s), we postulated that blocking hypothalamic AMPK could influence ghrelin-stimulated GH secretion by altering one of the above mechanisms. Although we cannot discard that AMPK at other extrahypothalamic loci might be also implicated. In order to (indirectly) test the first possibility, we assessed GH responses to exogenously administered GHRH after the pharmacological inhibition of hypothalamic AMPK. The fact that GHRH-induced GH secretion was markedly inhibited indicates that, at least in part, the inhibitory effect exerted by functional impairment of AMPK is mediated through a non-GHRH dependent mechanism. Although definitive proof is yet lacking, the fact that GH responses to both GHRH and ghrelin were markedly suppressed strongly suggests a mechanism mediated by an increased somatostatinergic tone. Our argument is based on the generally accepted view regarding the neuroregulation of GH secretion. This model indicates that the pulsatile secretion of growth hormone (GH) from the anterior pituitary [38] is generated by the opposing actions of somatostatin (SS) and GH-releasing factor (GRF) [39]. SS secreted from neurons located mainly within the periventricular nucleus (PeN) inhibits the GH release [40] whereas GHRH secreted from neurons in the arcuate nucleus (ARC) stimulates its release. Taking into account that the blockade of AMPK blunted exogenous GHRH-induced GH secretion, the data suggest that the effect is mediated by an increased somatostatinergic tone. In this regard, it is noteworthy that data gleaned using different experimental models indicate that most of the metabolic signals (e.g., glucose and amino acids, such as arginine) appear to influence GH secretion by acting at the hypothalamic level through a somatostatin-dependent mechanism [41,42,43].

In any event, regardless of the mechanism involved, these findings add further relevance to the importance of hypothalamic AMPK in the control of different homeostatic processes, including the neuroendocrine control of the GH-axis. Interestingly, this effect appears to be quite specific, since LH responses to exogenously administered GnRH, namely the main elicitor of gonadotropin secretion [44], remained unaffected after blockade of central AMPK signaling. Noteworthy, this observation does not refute the possibility of regulatory actions of AMPK on the hypothalamus–pituitary–gonadal axis, at other levels and/or in other physiological and experimental settings, as recently suggested by our data on the function of AMPK signaling in Kiss1 neurons in the metabolic control of puberty [21]. Yet, pituitary responsiveness to GnRH in terms of LH secretion seems to be independent of central AMPK.

One of the striking features of AMPK-dependent ghrelin orexigenic effect was that the activation of AMPK led to marked changes in hypothalamic lipid metabolism, as shown by the decreased hypothalamic malonyl CoA levels and an increased CPT1 activity [4,18]. In fact, blockade of CPT-1 activity was as effective as inhibition of hypothalamic AMPK in attenuating the orexigenic effect of ghrelin. Therefore, we anticipated that a similar mechanism could operate in terms of control of GH secretion. However, contrary to our original hypothesis, we found that etomoxir, at doses known to inhibit ghrelin-induced food intake [18], was unable to impair GHRH- or ghrelin-induced GH secretion in vivo. This indicates that although AMPK plays a critical and pivotal role on the two most relevant biological effects of ghrelin, namely, food intake and GH secretion, the molecular events triggered by hypothalamic AMPK for the control of each function are different. Further support for this possibility is reinforced by data showing that ghrelin-induced food intake is mediated, via AMPK, by a Sirt-1 dependent mechanism while GH secretion was not [45].

Besides the relevance of AMPK as a master sensor and regulator of energy homeostasis at the cellular level, compelling data have suggested that mTOR, and in particular mTORC1, a rapamycin-sensitive kinase, play a counterbalancing role to AMPK in the control of energy homeostasis. Furthermore, previous data have shown that inactivation of central mTOR blocks the positive effects of leptin on puberty onset, thus demonstrating that this pathway plays a critical role in the neuroendocrine control of key physiological/hormonal events [20]. In addition, there are some findings linking mTORC1 to several functional and biological effects of the GH axis [15]. A hypothesis to explain the alterations connecting GH and aging [46], involving decreased GH signaling, decreased IGF-1 levels, decreased mTOR and increased insulin sensitivity, have been put forward. In fact, some of the biological effects of GH appear to be mTORC1-dependent, since rapamycin blocks GH-induced incorporation of s35 methionine incorporation in target tissues [47].

Finally, this kinase also appears to play a critical role in somatotroph cells, since rapamycin induces G0/G1 cell cycle arrest in the related GH3 line [48,49]. All these findings led us to assess the role of mTOR on basal and stimulated GH secretion in vivo. Our data showed that the inhibitor of mTOR, rapamycin, does not modify either GHRH- or ghrelin-induced GH secretion. Thus, our data do not support a role for hypothalamic mTORC1 in the control of GH secretion, at least under this experimental setting. Nevertheless, future work assessing the impact of mTOR blockade in other physiological settings, such as puberty, is warranted in the light of the marked effects exerted by this kinase in puberty onset [20], as the period in the lifespan when many adaptative changes in GH secretion takes place. Intriguingly, despite our previous findings on the clear impact of inhibition of the central mTOR in pubertal progression in female rats, in our present study, no effect of central administration of rapamycin on GnRH-induced LH secretion was detected in adult male rats. Yet, this finding is not unexpected, since we have previously shown that the inhibitory effect of rapamycin on the gonadotropic axis is mainly exerted upstream of GnRH targets, namely in the Kiss1-expressing neurons, which are essential stimulators of GnRH neurons [50] and are inhibited by mTOR blockade [20].

In summary, this study documents for the first time an indispensable role of hypothalamic AMPK signaling in the control of GH responses to GHRH and ghrelin in vivo. The mechanism by which AMPK conducts this function is independent of hypothalamic lipid metabolism, since functional blockade of CPT-1 with etomoxir did not impair GH responses to these secretagogues. In addition, the specific importance of AMPK in the neuroendocrine control of GH secretion is highlighted by our finding that mTOR, the other master sensor and regulator of energy homeostasis, does not appear to play a similar role. On the basis of our present data, future studies assessing the function of hypothalamic AMPK dependent and non-dependent lipid signaling will allow us to dissect out in a more specific way the central mechanisms governing neuroendocrine function. This could explain the altered control of GH secretion in various conditions linked to altered energy balance and metabolic homeostasis.

## Figures and Tables

**Table 1:** Summary of the compounds used in the present study and detailed information about their actions, dosage and reference for dose selection.

Compound	Target/Action	Commercial Source	Dose (Ref No)
compound C (CC)	AMPK inhibition	P5499; Sigma Aldrich (St Louis, MO, USA)	10 μg [4,18]
etomoxir (ETOM)	CPT1 inhibition	E1905; Sigma Aldrich (St Louis, MO, USA)	10 μg [13,18]
rapamycin (RAPA)	mTOR inhibition	553210; Calbiochem (San Diego, CA, USA)	50 μg [15,20]
